# Supplementary material for: Pulsed Low-Frequency Magnetic Fields Induce Tumor Membrane Disruption and Altered Cell Viability
Source: Biophys J. 2020 Feb 18;118(7):1552–63. doi: 10.1016/j.bpj.2020.02.013 (PMC7136334; doi:10.1016/j.bpj.2020.02.013)

Biophysical Journal, Volume *118*

Supplemental Information

Pulsed Low-Frequency Magnetic Fields Induce Tumor Membrane Disruption and Altered Cell Viability

Christopher P. Ashdown, Scott C. Johns, Edward Aminov, Michael Unanian, William Connacher, James Friend, and Mark M. Fuster

SUPPORTING MATERIAL FOR:

Pulsed Low Frequency Magnetic Fields Induce Tumor Membrane Disruption and Altered Cell Viability

C.P. Ashdown[1,5] S.C. Johns[1,2] E. Aminov[6] M. Unanian[7] W. Connacher[6] J. Friend[6] and M.M. Fuster[1,2,3,4] *

[1] VA San Diego Healthcare System and [2] Veterans Medical Research Foundation, San Diego, CA, USA; [3] Department of Medicine, Division of Pulmonary & Critical Care, University of California San Diego, La Jolla, CA, USA; [4]Glycobiology Research and Training Center, University of California San Diego, La Jolla, CA, USA; [5] Division of Biological Sciences, University of California San Diego, La Jolla, CA, USA; [6]Dept of Mechanical and Aerospace Engineering, University of California San Diego, La Jolla, CA, USA; [7]School of Electrical Engineering, Columbia University, New York, NY, USA

SUPPLEMENTAL FIGURE LEGENDS

Supplemental Figure S1. Pulsed magnetic field exposure and photomicrograph of A549 monolayers showing density of cells at which exposure to magnetic fields was carried out. (A) A DC power supply was used to generate magnetic fields through a commercial solenoid in the milli-Tesla (20 mT maximum) range, with oscillating frequencies (50 Hz and 385 Hz) by interrupting the voltage input with an appropriate circuit for oscillations (shown to right). Cells plated as monolayers in wells of a 96-well plate (shown over the solenoid platform) were exposed to magnetic fields fluxing orthogonal to the plate-bottom. (B) View looking downward into plate-wells positioned over the solenoid platform, with configuration of 4 wells (marked in red) positioned over solenoid central-core (edge of uniform-grey metallic platform marked with a “P”) that uniformly exposed cells plated on the 4 marked wells to the orthogonal magnetic field emanating vertically from the central platform. (C) Typical appearance of sub-confluent A549 cells (at ~ 80% confluence in this photomicrograph) growing as monolayer within a well at time of pulse-field exposure. Bar represents 100 μm. Pre-experiment growth of all cells to approximately 80% confluence was carried out to ensure uniform and comparable monolayer conditions for membrane integrity and cell viability studies.

Supplemental Figure S2. Pulsed magnetic field associated reduction in membrane integrity of LLC tumor cells. Magnet-exposed and control LLC cells were measured using an intracellular-protease-leak detection assay, and graph shows mean protease release signal of magnet-exposed cells normalized to that of control cells (**P*=0.02 for difference between the means; paired T-test with means representing average and SD; n=5 experiments).

Supplemental Figure S3. Dose response plot showing relative magnet-induced protease release (normalized to max response) as a function of magnetic field amplitude. To assess for the relative degree of altered membrane integrity/ protease leak as it depends on magnetic field strength, 10 min exposures of A549 monolayers to sequential 50 Hz/ 385 Hz pulsed magnetic fields at room temperature were carried out, with measurement of dead-cell protease release values in magnet-exposed versus control cells. The increase in protease release by magnet-exposed cells over that of control cells (as a percentage of control protease release) was calculated and plotted for a scale of increasing magnetic field strengths, and normalized to the value at the maximum field strength (20 mT) achievable using the oscillating solenoid system illustrated in Supplemental Figure S1. The means +/- SD for n=3 experiments at each field strength are shown in the histogram (**P*=0.03; ***P*=0.01; One Sample T-test for positive magnet-induced protease release over that of control).

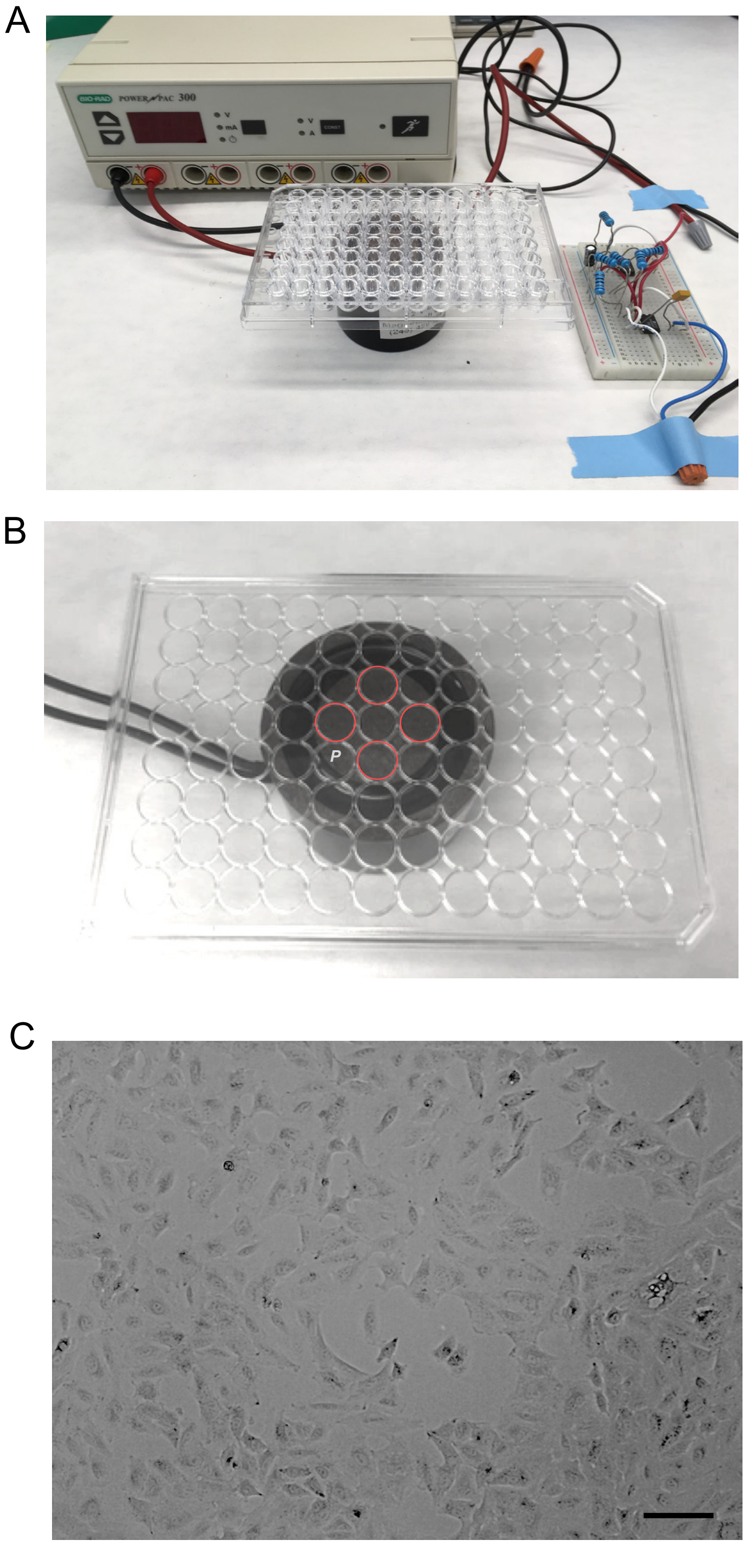
A
BIO-RAD
POWER PAC 300
B
P
C

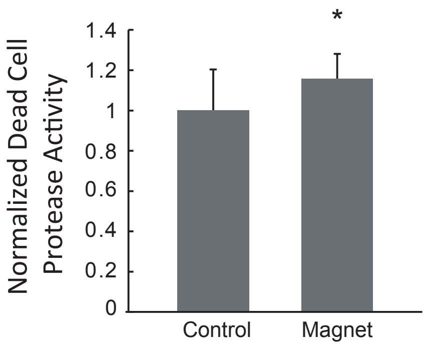
*
1.4
1.2
1
0.8
0.6
0.4
0.2
0
Normalized Dead Cell Protease Activity
Control
Magnet

Supplemental Figure S3

Ashdown, et al.

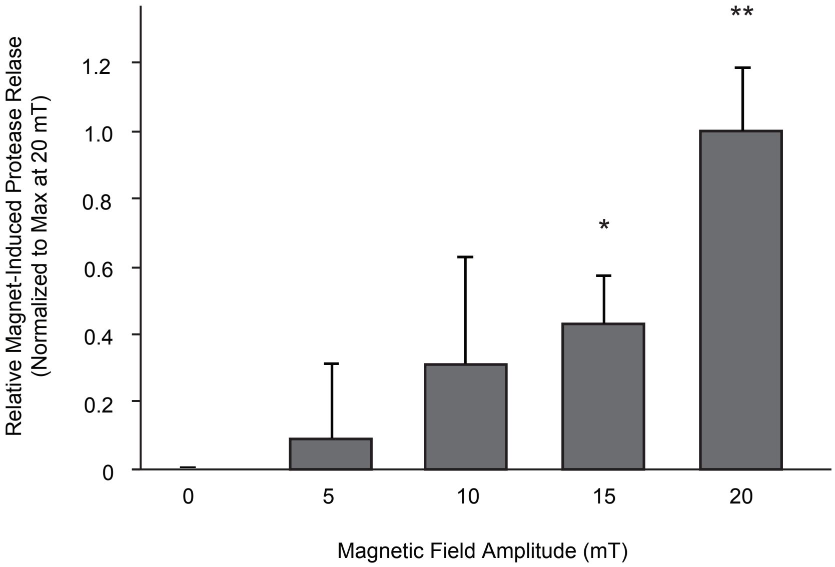

Supplement: Document S1. Figs S1–S3 [file mmc1.pdf]
