# Supplementary material for: Pulsed Low-Frequency Magnetic Fields Induce Tumor Membrane Disruption and Altered Cell Viability
Source: Biophys J. 2020 Feb 18;118(7):1552–63. doi: 10.1016/j.bpj.2020.02.013 (PMC7136334; doi:10.1016/j.bpj.2020.02.013)

Biophysical Journal
Article

Biophysical Society

Pulsed Low-Frequency Magnetic Fields Induce Tumor Membrane Disruption and Altered Cell Viability

Christopher P. Ashdown,[1,5] Scott C. Johns,[1,2] Edward Aminov,[6] Michael Unanian,[7] William Connacher,[6] James Friend,[6] and Mark M. Fuster[1,2,3,4,*]

[1]VA San Diego Healthcare System and [2]Veterans Medical Research Foundation, San Diego, California; [3]Department of Medicine, Division of Pulmonary & Critical Care, [4]Glycobiology Research and Training Center, [5]Division of Biological Sciences, and [6]Department of Mechanical and Aerospace Engineering, University of California, San Diego, La Jolla, California; and [7]School of Electrical Engineering, Columbia University, New York, New York

ABSTRACT Tumor cells express a unique cell surface glycocalyx with upregulation of sulfated glycosaminoglycans and charged glycoproteins. Little is known about how electromagnetic fields interact with this layer, particularly with regard to harnessing unique properties for therapeutic benefit. We applied a pulsed 20-millitesla (mT) magnetic field with rate of rise (dB/dt) in the msec range to cultured tumor cells to assess whether this affects membrane integrity as measured using cytolytic assays. A 10-min exposure of A549 human lung cancer cells to sequential 50- and 385-Hz oscillating magnetic fields was sufficient to induce intracellular protease release, suggesting altered membrane integrity after the field exposure. Heparinase treatment, which digests anionic sulfated glycan polymers, before exposure rendered cells insensitive to this effect. We further examined a non-neoplastic human primary cell line (lung lymphatic endothelial cells) as a typical normal host cell from the lung cancer microenvironment and found no effect of field exposure on membrane integrity. The field exposure was also sufficient to alter proliferation of tumor cells in culture, but not that of normal lymphatic cells. Pulsed magnetic field exposure of human breast cancer cells that express a sialic-acid rich glycocalyx also induced protease release, and this was partially abrogated by sialidase pretreatment, which removes cell surface anionic sialic acid. Scanning electron microscopy showed that field exposure may induce unique membrane "rippling" along with nanoscale pores on A549 cells. These effects were caused by a short exposure to pulsed 20-mT magnetic fields, and future work may examine greater magnitude effects. The proof of concept herein points to a mechanistic basis for possible applications of pulsed magnetic fields in novel anticancer strategies.

SIGNIFICANCE The ability to noninvasively alter the membrane integrity of cancer cells through unique electromagnetic wave applications has appealing therapeutic translational potential. Pulsed magnetic fields, which may penetrate human tissues "in the spirit of MRI," are enticing as possible anticancer therapeutic strategies. Our findings herein suggest the possibility that pulsed magnetic fields may selectively alter cancer cell membranes and viability without the use of ionizing radiation or delivery of molecular or cytotoxic agents. Depending on the ultimate magnitude of effects, it is possible that such fields could be applied as adjuvant therapies when paired with standard anticancer treatment. With further research, such fields might also be harnessed to facilitate delivery of anticancer agents across tumor cell membranes.

INTRODUCTION

A small body of research shows that magnetic field exposures may modulate tumor cell behavior in vivo (1–4). Previous studies have shown some success in treating rodent tumors with magnetic fields in the millitesla (mT) range and with frequencies far under 500 Hz (3–8). However, the cellular mechanisms and the nature of the unique effects on tumor cells remain poorly understood. A particularly intriguing cellular domain that may be vulnerable to electromechanical coupling through novel application of electric field or magnetic flux oscillations is the glycocalyx, a dense complex-carbohydrate layer that decorates proteins on the mammalian plasma membrane (9). The glycocalyx is endowed with a dominant negative charge composition due to anionic sugars (e.g., sialic acid modifications and/or

Submitted July 15, 2019, and accepted for publication February 10, 2020.

*Correspondence: mfuster@ucsd.edu

Editor: Cynthia Reinhart-King.

https://doi.org/10.1016/j.bpj.2020.02.013

This is an open access article under the CC BY license (http://creativecommons.org/licenses/by/4.0/).

sulfated sugars) that may be greatly upregulated in unique pathologic states, including neoplasia (10). Theoretically, although the frequency of oscillation may critically couple to mechanical resonance if selected appropriately, a key parameter that is relatively independent of the frequency of pulses may be the rate of change in the magnetic field (dB/dt) with each pulse (rise time for duty cycle). Indeed, some studies demonstrated effects using frequencies as low as 1–2 Hz (7,8,11), with the biological effects ultimately depending more on a sufficiently narrow pulse width (<200 ms) than the pulse frequency. This means that the exact frequencies used may be less important as long as the magnetic system is able to "rapidly" respond to changes in driving current in the case of a coil or solenoid system.

In general, cancer cells express higher levels of negatively charged glycosaminoglycans (GAGs) and glycoproteins than that of normal differentiated cells (10,12). Both GAGs and glycoproteins have been implicated in immunosuppressive mechanisms and can facilitate metastatic functions through binding interactions with unique receptors (10,12,13). However, the ability to interact with these specific molecules with physical stimuli for the purpose of antitumor therapy is an area that needs further exploration. Although there have been some studies investigating antitumor effects of external whole-animal magnetic fields using in vivo mouse models (3,4), to the best of our knowledge, there is no literature examining how these effects are initiated at the cellular level, and only minimal work characterizing downstream biological effects (2,5,14).

In theory, if dB/dt is high enough, applying a magnetic field pulse should generate a torsional electromotive force (EMF) on any charge-carrying elements of the cell surface, so long as the charge density is high enough. This effect may operate through Faraday's law of induction. Indeed, neuronal charge distributions may be driven by transcranial magnetic stimulation to affect neuronal function via EMFs generated by magnetic induction (15). EMF is defined as the negative cross product of the change in flux of the magnetic field across a defined area ($A \times dB/dt$). Therefore, in monolayers of tumor cells, with applied pulsed magnetic fields, one might potentially drive cell surface molecular forces through EMFs conducted over relatively broad cellular areas (100 cells wide or greater). Greater density of confluent negative surface charge could theoretically increase the force on charged cell surface molecules in the EMF path. In addition to this, if the change in magnetic flux (via change in dB/dt) is fast enough, and the molecular charge density is high enough, the resulting EMF may have torsional or disruptive effects on the glycocalyx and possibly on cell membrane integrity. It is also possible that such EMFs may disrupt ion channel homeostasis on tumor cell membranes. Any or all of these effects might alter downstream tumor cell viability and growth.

The human lung carcinoma cell line A549, like other carcinoma cells, is known to overexpress the sulfated GAG heparan sulfate (HS) (12,16,17); and the mouse Lewis lung carcinoma (LLC) cell line, an aggressive, poorly differentiated mouse lung cancer cell line, is endowed with a similarly charged glycocalyx (18). In addition, certain other carcinoma cells, such as the human breast cancer line MDA-MB-231, are endowed with heavy expression of the anionic sialic acid as a terminal complex-carbohydrate "capping" sugar on tumor membrane glycoproteins. These cells may be tested in monolayer in vitro arrays over magnetic fields in the laboratory. Herein, we demonstrate the effects of pulsed magnetic fields on tumor cell membrane integrity, viability, and growth in human lung cancer cells as a model; and apply targeted proof-of-concept assays to other cells with a focus on both mechanistic insights and an eye toward unique therapeutic application.

MATERIALS AND METHODS

Cell culture and preparation

A549 cells were obtained from American Type Culture Collection (ATCC; Manassas, VA). They were grown at 37°C in Ham's F-12K medium supplemented with 10% FBS and 1% PenStrep (GibCo, Waltham, MA). Cells were subcultured once they reached 80% confluency using 0.5% trypsin, 0.2% EDTA solution to lift cells, and plated into either a 96-well (96w) or 12-well (12w) format, depending on the assay being used. Cells were plated at 5000 cells per well (96w plates) or at 25,000 cells per well (12w plates) and were allowed to grow in culture overnight before being exposed to experimental conditions. As a non-neoplastic nonepithelial cell line from the human lung microenvironment, human lung lymphatic microvascular endothelial cells (hLEC; Lonza, Basel, Switzerland) were grown at 37°C in Endothelial Cell Growth Medium-2 (EGM-2V; Lonza) and subcultured into either a 12w or 96w format, depending on the assay used. Cells were seeded at 5000 cells per well (96w plates) or 50,000 cells per well (12w plates) using the subculture ReagentPack (Lonza) for both. LLC cells (ATCC) were grown at 37°C in Dulbecco's Modified Eagle Medium (DMEM) high-glucose media supplemented with 10% fetal bovine serum (FBS) and 1% PenStrep (GibCo). MDA-MB-231 human breast carcinoma cells were cultured in DMEM medium with 10% FBS and 1% PenStrep (Gibco). All cells were harvested from culture plates using an Accutase solution (Corning, Corning, NY) to lift cells, and plated into 96w plates. Cells were plated at 5000 cells per well in the 96w plates and were allowed to grow overnight before being exposed to experimental conditions. Care was taken to subculture LLC cells before they self-detached. For 96w-based membrane integrity and short-term viability studies, cells were allowed to grow to ~80% confluency before magnetic field exposure.

Magnetic field exposure and characteristics

The experimental source consisted of a solenoid magnet (#R-2016-12; Magnatech) interrupted by a circuit and connected to a standard power supply (BioRad Power Pack, 10 V DC current; Bio-Rad, Hercules, CA) to allow for pulsing fields (20-mT maximum orthogonal to cell monolayer at surface) at discrete frequencies (50 and 385 Hz). The magnet performance allowed for dB/dt in the millisecond range. This was ~10 ms for the specific solenoid and driving conditions we employed, theoretically allowing for duty cycle completion with each period during the 50-Hz-frequency application. The maximum strength of the magnet at the bottom of a well in the overlying plate was confirmed using a Gauss meter (PCE Instruments, Southampton, UK), measured 20 mT maximum amplitude, and generated with flux orthogonal to the well's bottom surface, where

monolayer cells were grown. On any day of exposure and measurement, for any given trial, the plate with cell monolayers was placed at room temperature on top of the magnet, and applied fields were delivered sequentially for 5 min each (50 Hz followed by 385 Hz), whereas control cells plated under equivalent conditions and incubated in parallel to magnet-exposed cells remained in the absence of a magnetic field under otherwise identical conditions. The two frequencies we used were applied arbitrarily as two low-frequency variations within the order of magnitude of those used in previous studies that specifically employed low-frequency (<500 Hz) fields. For any given plate, at the time of magnetic field exposure, plated cells were ~80% confluent. Fig. S1 illustrates the experimental setup along with a 20× objective microscope view of plated cells at time of exposure.

For a magnetic field "dose" (strength)-response test, a dose curve was established using 1-mm lifters that created a stepwise increase in distance from the solenoid base. Standard microscope slides (25 × 75 × 1 mm) were stacked to create a platform for raising or lowering the plated cells above the magnetic field. A field-strength curve was created as follows: 20 mT = maximum amplitude at solenoid surface, 15 mT measured at 2 mm above the surface; 10 mT measured at 5 mm above the surface, and 5 mT at 9 mm above the surface. A Gauss meter was used to confirm magnetic field strengths for plated cells on well bases at the stated heights.

Cell membrane integrity assays

A luminescence-based kit (AAF-Glo cytotoxicity; Promega, Madison, WI) was used according to the manufacturer protocol for 96w application to assay for pulsed-magnetic-field-induced altered membrane integrity (cytotoxicity), as measured by protease release into the media. Cells were seeded in 96w plates and cultured for 24 h before magnetic field exposure to allow for plate attachment and monolayer establishment. After magnet exposure, in addition to acquisition of experimental protease levels reflecting cytotoxicity for each well, and to quantitatively assess stability in total cell load well to well, we applied digitonin. The digitonin exhaustively permeabilizes cell membranes to yield maximal protease release, according to the manufacturer's specifications. In most experiments, variation in cell load was less than 10%, and values were indexed to digitonin values, reflecting maximum achievable cytotoxicity. In one group of experiments using A549 cells, measurements demonstrated especially high stability (variation less than 5%), and thus raw values for protease release were reported. Moreover, similar data were obtained for MDA-MB-231 cells, whether reported as means of raw protease release values (reported as "Dead Cell Protease Activity" in Fig. 4 *A*) or values indexed to digitonin.

Glycan enzymatic digestion

Heparin lyase III (H'ase III) was a kind gift from the laboratory of Dr. J. Esko. H'ase III was used at 2.5 mU/ml at 37°C for 1 h to exhaustively digest HS on cell surfaces. Cells were washed with phosphate-buffered saline, followed by the addition of fresh Ham's F-12K medium before subsequent experimental exposures. Testing of fluorescent fibroblast growth factor-2 (FGF-2) binding to the cell surface was carried out by incubating biotinylated FGF-2 for 30 min, followed by washing, streptavidin-phycoerythrin labeling, and flow cytometry. This was carried out as published (19) to assess cell surface HS ligand binding capacity, wherein FGF-2 is used as a classic probe to estimate the relative load of HS on the cell surface by flow cytometry (20). Cells treated with and without H'ase III were tested for FGF-2 binding as a reporter of H'ase III-mediated HS digestion efficacy. For sialic acid digestion from MDA-MB-231 cells, plated cells were treated with and without 5 mU/mL of AUS sialidase (sialidase from Artrhobacter ureafaciens #EC-32118; EY Labs, San Mateo, CA) diluted in buffer (0.05 M HEPES, 1 mM $CaCl_2$, 1 mM $MgCl_2$; pH 6.9) for 1 h at 37°C. Cells were exposed to the magnetic field, with cytotoxicity (i.e., disruption in cell membrane integrity) assayed as previously described. The effects of AUS treatment were confirmed by flow cytometry of cells treated ± AUS using the lectin markers Biotinylated Maackia Amurensis Lectin (MAL-II, Vector B-1265) or Biotinylated Sambucus Nigra Lectin (SNL, Vector B-1305), which were incubated (at 1:100 dilution for 1 h at 4°C) before labeling with streptavidin-phycoerythrin for flow cytometry.

Short-term cell viability assays

To assess short-term functional effects of pulsed magnetic fields that affect cell growth potential, metabolic-based cell viability assays were employed. Cells were seeded in 96w plates and cultured overnight for plate attachment and monolayer establishment. On the next day, magnet exposure was carried out, and viability was measured 4 h postexposure using a luminescence-based ATP production assay according to the manufacturer's protocol (CellTiter-Glo; Promega).

Cultured cell proliferation assays

Beginning at the time of magnet exposure, 24 h after cell seeding into 12w plates, cell growth was measured through daily cell counts harvested from replicate plates over a 4-day time period. On any given day, 0.5% trypsin/0.2% EDTA solution was used to harvest all cells from the plate, and cells were counted through the use of an automated hemocytometer (Countess II; Life Technologies, Carlsbad, CA). (For one of four A549 trials, a manual hemocytometer was used with appropriate cell counting calibration.)

Scanning electron microscopy

A549 cells were seeded on indium/tin oxide (ITO)-coated coverslips (2spi 6468-AB) pretreated with 0.1 mg/mL Poly-D-Lysine and grown in the 37°C incubator overnight in standard growth medium. Cells were then exposed to the 10-min pulsed magnetic field as previously described and immediately fixed in 2.5% glutaraldehyde in 2 mM $CaCl_2$ and placed on ice for 1 h. A non-magnet-exposed control sample was flash treated for 1 min with 0.1% Triton X-100 at room temperature after fixation as a known membrane-disruption stimulus. Cells were then rinsed in 0.1 M cacodylate buffer, with five washes at 3 min each. Postfixing was done in 1% osmium tetroxide for 30 min on ice, followed by rinsing in double-distilled H_2O. Coverslips were dehydrated stepwise in graded ethanol series followed by critical point drying using standard CO_2 exchange. Before imaging, all samples were sputter coated with a gold-palladium source (E5100; Polaron Instruments). Images were acquired on a Zeiss scanning electron microscope (Gemini FE-SEM) at 3.0 kV. Magnification for multiple photomicrographs used for cell surface analysis was 58,840×.

Statistics

Averages and standard deviations for measurements are graphed, with comparisons of means as indicated. For most data analyses (e.g., mean values for cell cytotoxicity assays, short-term cell viability assays, and cell proliferation experiments), comparisons of means were carried out using a Student's *t*-test, with appropriate application for paired or unpaired data, wherein statistical significance for any differences was assumed at a level of $p < 0.05$. For *p*-values that achieved a near-significance value (i.e., $p = 0.06$ in two instances), the exact value ("p=0.06) was indicated over the appropriate data on the figure, with reporting in the legend. On occasion, a one-sample *t*-test was applied in which a sample of observations was compared to a single specific mean (e.g., testing the set for significant difference from 0 in a specific direction). In other cases, if the data distribution indicated the need for plotting median with interquartile ranges, the use of an appropriate nonparametric statistic, such as the Mann-Whitney U Test for comparing unpaired data, was applied to assess for significant differences between groups.

RESULTS

Exposure of A549 monolayer cells to pulsed magnetic fields was sufficient to alter membrane integrity, and sulfated glycans play an important role in mediating this effect

To measure membrane integrity alterations induced by the pulsed magnetic fields, magnet-exposed and control A549 cells were measured immediately after exposure by assaying for membrane leak (cytotoxicity) using an intracellular protease leak detection assay. Fig. 1 *A* shows protease release signal of control versus magnet-exposed cells. Magnet-exposed cells showed a mean 26% increase in cytotoxicity signal compared to basal protease release by resting control cells at room temperature over the same period (*$p = 0.02$ for difference; paired *t*-test). As a separate proof of concept using a distinct tumor cell line, we noted similar membrane integrity effects using the LLC mouse lung carcinoma cell line, which showed a 15% increase in cytotoxicity relative to normalized control (*$p = 0.02$ for difference; Fig. S2). To investigate a possible relationship between glycocalyx charge and membrane alteration effects, we explored whether the sulfated GAG heparan sulfate (HS), a major contributor to glycocalyx anionic charge, might be responsible for magnetic-induction-driven cytotoxicity effects. The endoglycosidase heparin lyase III (H'ase III) was used to exhaustively digest HS from cell surfaces, with washing before magnet exposure. Strikingly, we found that H'ase III-treated cells were insensitive to the pulsed magnetic field (Fig. 1 *B*). In particular, we found no significant difference in cytotoxicity between untreated control cells and H'ase III-treated magnet-exposed cells. Furthermore, H'ase III by itself did not alter cytotoxicity (compared with "negative control" in Fig. 1 *B*). To verify that H'ase III successfully destroyed HS chains on A549 cells, flow cytometry was used pre- and post-H'ase III treatment to assay for FGF-2 binding, a validated method for assessing the relative abundance of glycocalyx HS present on the cell surface. Fig. 1 *D* shows the results of flow cytometry, which demonstrate a significant pool of HS-associated charge on the A549 surface, as assessed by the extent of FGF-2 binding to cell-surface anionic sulfated HS chains. Binding was nearly completely ablated by H'ase III treatment.

Pulsed magnetic field exposure inhibits viability and growth of A549 carcinoma cells

Short-term cell viability of A549 cells was measured after a 4-h cell culture incubation period after magnetic field exposure using an assay that quantifies ATP levels to measure overall metabolic activity (Fig. 2 *A*). In addition to nonexposed cells as healthy negative controls, we employed treatment with staurosporine, a protein kinase inhibitor used to induce apoptosis and inhibit metabolic activity (21), as a positive control for inhibition of cell viability. Staurosporine significantly reduced A549 viability (*$p = 0.03$ for difference with that of negative control); and pulsed magnetic field exposure also reduced cell viability to a magnitude comparable to that of staurosporine treatment, with mean viability of magnet-exposed cells reduced by 28% ($p = 0.06$ for difference with that of negative control). After discovery that pulsed magnetic field exposure affects metabolic activity, cell proliferation in culture was measured by counting cell number over multiple days. Fig. 2 *B* shows growth curves with mean daily cell counts, and demonstrates a significant difference in proliferation between cells that were magnet-exposed on day 0 and that of control cells by day 4, with mean growth of magnet-exposed cells reduced by 26% (*$p = 0.05$ for difference relative to control). Cell growth in magnet-exposed cells was noted to splay from control growth over the 4-day period, indicating that a relatively short magnet exposure (10 min) is sufficient to impair continued cell growth in culture over several days.

Nontumor human lymphatic endothelial cells are insensitive to pulsed magnetic field effects on membrane integrity and growth

We examined the effect of pulsed magnetic field exposure on normal (non-neoplastic) host cells that may be found in a lung carcinoma. In particular, we applied pulsed magnetic fields to human lymphatic endothelial cells (hLECs), which would typically be found in the microenvironment of a lung adenocarcinoma, from which A549 cells were originally derived. Magnet-exposed and control hLECs were plated and assayed for cytotoxicity under the same conditions as those of A549 cells. Interestingly, we found that for hLECs, there was no significant difference between protease release from magnet-exposed versus control cells (Fig. 3 *A*; $p = 0.33$ for comparison of means, and compare with Fig. 1 *A* for A549 cells). Fig. 3 *B* shows growth curves of mean daily cell counts for magnet-exposed and control hLECs, and demonstrates no difference in cell proliferation over the entire 4-day growth period between groups. Flow cytometry (Fig. 3 *C*) assaying for FGF-2 binding suggests a markedly lower degree of sulfated HS on hLECs as compared with A549 cells, with rightward shift in flow histogram relative to control as a measure of cell surface HS sulfation, which was overall reduced in comparison to that of A549 cells (compare log-shift in FGF-2 binding relative to controls in Fig. 3 *C* to that of Fig. 1 *D*). These findings may point to the selective importance of HS in the tumor glycocalyx as a mediator of the effects of pulsed magnetic fields on membrane integrity and viability.

Exposure of MDA-MB-231 monolayer cells to pulsed magnetic fields was sufficient to alter membrane integrity, and effects are partially abrogated by elimination of cell surface sialic acid

Given that sialic acid capping on the glycan termini of glycoproteins is upregulated in many carcinomas and serves as

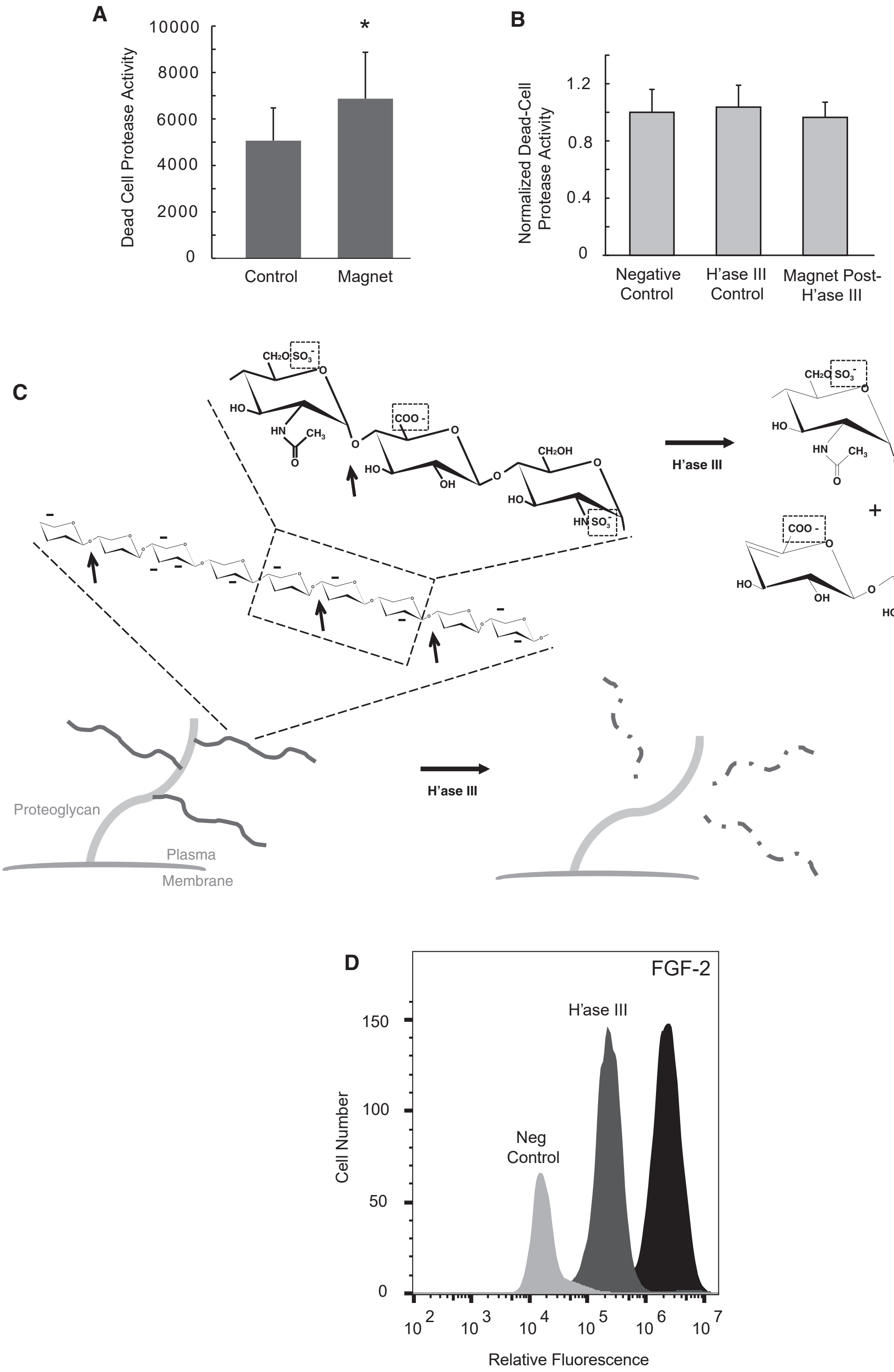

FIGURE 1 A pulsed magnetic field induces an HS-dependent plasma membrane leak in A549 carcinoma cells. (*A*) Shown is membrane integrity from magnet-exposed and control A549 cells showing relative protease release signal of control versus magnet-exposed cells (*$p = 0.02$ for difference between the means; paired *t*-test; means represent average and SD among $n = 6$ experiments). (*B*) The effect of heparin lyase III (H'ase III) pretreatment on altered membrane integrity of magnet-exposed and control A549 cells. Bars represent normalized means for cytotoxicity. The leftmost bar shows data for untreated control cells. Bars on the right show data for cells that were pretreated with H'ase III (means between groups were NS; $n = 8$ experiments). (*C*) Schematic demonstrating the structure of a model HS proteoglycan on the cell surface, with insets demonstrating charged glycan polymer chain (*left side*; charged sulfate and carboxyl groups in dotted boxes on detailed glycan inset, with typically multiple 40-mer to 80-mer HS polymer chains attached to the core protein).

(legend continued on next page)

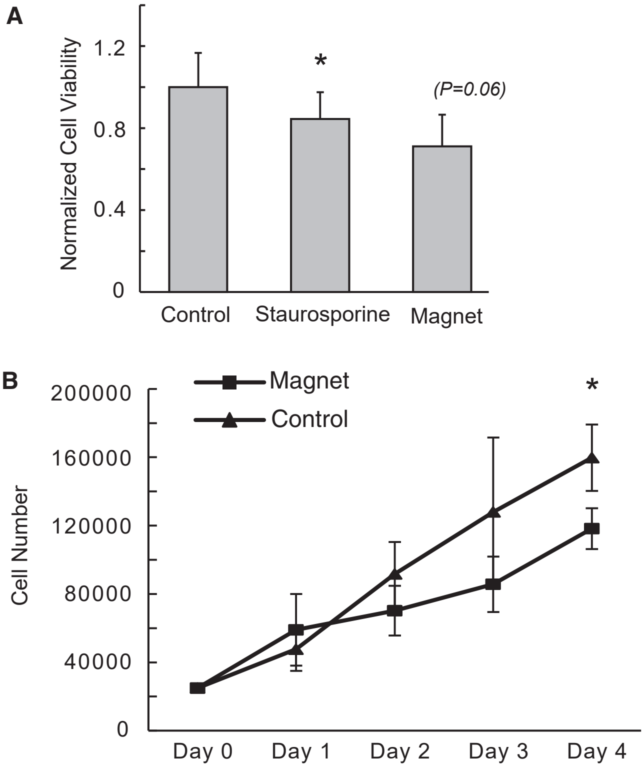

FIGURE 2 Pulsed magnetic field conditions that induce membrane leak alter cell viability in A549 cells. (*A*) Magnet-exposed and control A549 cells were assayed for short-term viability at 4 h following field exposure. The bars in the graph represent normalized means for ATP measurements, indicating cell viability. Controls include non-magnet-exposed cells (*leftmost bar*) and staurosporine-pretreated non-magnet-exposed cells (apoptosis inducing control; *center bar*). The rightmost bar represents magnet-exposed cells (*$p = 0.03$ for difference between control and staurosporine; $p = 0.06$ for difference between control and magnet; paired *t*-test; n = 4 experiments). (*B*) The effect of pulsed magnetic field exposure on long-term A549 cell proliferation. Magnet-exposed and control A549 cells (plated on day 0 at 25,000 cells per well) were harvested daily over a period of 4 days after magnet exposure on day 1. Graph reveals overall splay in growth rates of control versus magnet-exposed cells over the growth period. Mean reduction in cell number at day 4 as a result of magnet pretreatment was 26% (*$p = 0.05$; n = 4 experiments).

another route by which the glycocalyx becomes endowed with anionic charge, we employed MDA-MB-231 human breast cancer cells, which are endowed with a relatively high cell surface sialic acid content, to examine whether eliminating this glycan alters the effect of the pulsed magnetic field on membrane integrity. As noted in A549 cells (Fig. 1), MDA-MB-231 tumor cells also succumb to significant protease release as a result of the 10-min pulsed field exposure (Fig. 4 *A*, left "buffer-only" bars). Interestingly, for MDA-MB-231 cells pretreated with AUS sialidase, which digests all possible sialic acid linkages (i.e., α2,3- or α2,6- to internal galactose or α2,8- to internal sialic acid), whereas pulsed field exposure results in a significant degree of protease release (Fig. 4 *A*, right "post-AUS" bars), enzymatic clearance of terminal sialic acid from the cell significantly inhibits the relative degree of protease release (compare buffer-only magnet response to post-AUS magnet response in Fig. 4 *A*; **$p = 0.021$ for difference). The partial abrogation of magnet-induced protease leak as a result of sialic acid removal by AUS (Fig. 4 *B*) implies a significant role for this charged glycan in mediating the magnetic field effects on protease release by MDA-MB-231 cells. Flow cytometry using fluorescent lectins revealed that sialic acid was markedly reduced by AUS sialidase (Fig. 4 *C*), with a 5-fold reduction in α2,3-linked sialic acid (as assessed by MAL-II lectin binding) and a >50% reduction in α2,6-linked sialic acid (as assessed by SNL lectin binding).

The plasma membrane of A549 cells demonstrates patterns of increased "rippling" and nanopore formation as a result of pulsed magnetic field exposure

Immediately after exposure of A549 cell monolayers to the 10-min sequential 50/385-Hz pulsed magnetic field, we carried out scanning electron microscopy (SEM) with analysis of cell surface 58,840× photomicrographs, which revealed sparsely distributed pores in the range of 20–50 nm in diameter on the surfaces of both control cells and magnet-exposed cells, and which appear to be most consistent with likely caveolae or endocytotic events (Fig. 5 *A*; bar = 100 nm). Those have been reported in that size range on the cell surface (22). Smaller "nanopores" in the <20-nm-diameter range, however, appeared to be more frequently found on the surface of cells that were exposed to the pulsed magnetic fields (compare upper versus middle pairs of panels in Fig. 5 *A*, showing representative images, and noting smaller pores to right of while asterisks on magnet-exposed cells). As an additional membrane-disruption control, cells exposed to a short 0.1% Triton exposure (1 min postfixation) demonstrated a high frequency of holes with a wide dispersion in sizes, including conglomerate holes appearing as "moth-eaten" defects (Fig.5 *A*, lower panel). Quantifying nanopores in the <20-nm size range among multiple SEM fields among the different conditions confirmed a greater proportion of such pores in cells that

The effect of endoglycosidase H'ase III on digestion of the chain at multiple points is shown (i.e., at glucosamine-uronic-acid linkage, in which uronic acid is relatively unsulfated/uncharged; *arrows*). This fragments HS chains with essentially glycan-denuded (and charge-denuded) proteoglycan core proteins (shown to *right* of H'ase III *arrow*), which are unable to confer forces (and thus torque) by pulsed-magnet-induced (dB/dt) EMFs at the point of core protein attachment to the membrane. (*D*) Flow cytometry analysis of FGF-2 binding on A549 cells and the effect of H'ase III on FGF-2 binding. The rightmost histogram shows FGF-2 binding in untreated cells, with leftmost histogram as negative control; and the middle histogram shows FGF-2 binding to A549 cells post-treatment with H'ase III.

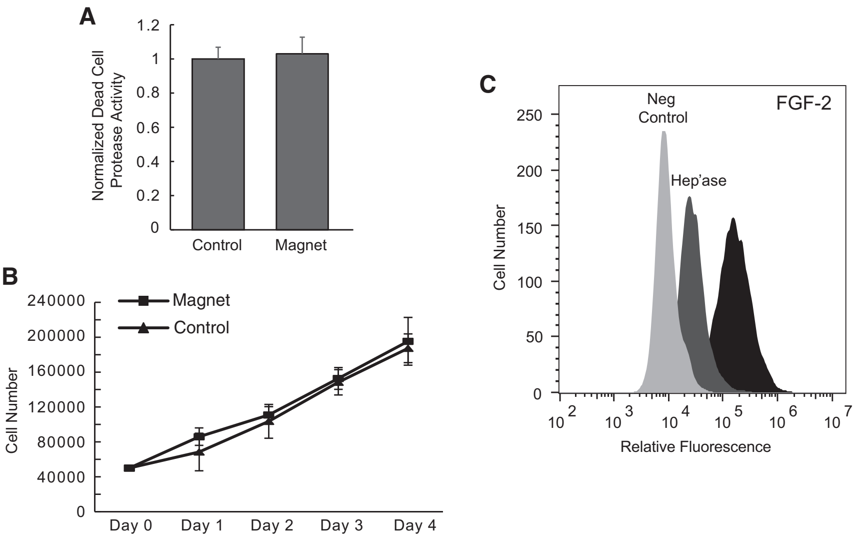

FIGURE 3 Membrane integrity and growth of non-tumor human lymphatic endothelial cell monolayers are insensitive to pulsed magnetic field treatment. (*A*) Membrane integrity of magnet-exposed and control hLECs showing normalized protease release signal between the two groups ($p = 0.327$ for difference between the means; paired *t*-test with means representing average and SD among $n = 4$ experiments). (*B*) Effect of pulsed magnetic field exposure on long-term hLEC proliferation. Magnet-exposed and control hLECs (plated on day 0 at 50,000 cells per well) were harvested daily over a period of 4 days after a single magnet exposure on day 1 (difference in daily cell counts between control and magnet exposure was NS). (*C*) Flow cytometry analysis of FGF-2 binding on hLEC cells and the effect of H'ase III treatment on FGF-2 binding. The rightmost histogram shows FGF-2 binding to untreated cells with leftmost histogram as negative control. The middle histogram shows FGF-2 binding following H'ase III treatment.

were pulse-field exposed (Fig. 5 *B*). A separate unique finding after the pulsed field exposure is a relatively high degree of "ridging" or parallel-running lines on the membrane of magnet-exposed A549 cells (Fig. 5 *A*, white arrowheads, middle panels; quantified in Fig. 5 *C*). This was not apparent in either control unexposed cells or Triton-pulsed cells.

DISCUSSION

Our data show that the application of a pulsed magnetic field is sufficient to induce altered membrane integrity on tumor cells, which have a glycocalyx that is rich in proteoglycans expressing sulfated GAG chains. The fact that tumor cell lines were susceptible to this physical stimulus is encouraging and hints that a pulsed magnetic field could have membrane-integrity-altering effects in a wider range of cancer cell types, provided that they display a similar glycocalyx charge composition. Furthermore, it is striking to find that removing glycocalyx charge in A549 cells, specifically via heparinase pretreatment to destroy HS GAG chains, is sufficient to protect the membrane from protease leak/disruption by the pulsed magnetic field stimulus. This suggests that the pulsed field is interacting with HS GAGs, possibly through electromagnetic induction, as they are abundant charged elements of the tumor cell glycocalyx. Interestingly, non-neoplastic primary cells typically found in the lung cancer microenvironment (i.e., host lymphatic endothelial cells) showed no integrity alteration upon exposure to the same pulsed magnetic fields. This suggests a tumor-selective effect of the pulsed field under the tested conditions. More sophisticated analyses of GAG composition of cancer cells versus noncancerous lines may shed light on the importance of sulfated and charged GAGs as a uniquely susceptible glycocalyx molecular target for pulsed magnetic fields, but this would need separate dedicated studies. Moreover, while studying how variation in all parameters of our system is beyond the scope of our proof of concept herein, we did note that varying magnetic field strength up to the maximum achievable in our solenoid system (20 mT) resulted in a steep rise in dead-cell protease leak up to the 20-mT maximum (Fig. S3). In the spirit of searching for a dose response, in analogy to establishing a "drug dose" ED50 or IC50, we would need to boost to higher magnetic field strengths (>20 mT) to establish such values: as such, although our current equipment cannot achieve this, such work would be of interest as dedicated studies with advanced technical systems. In the same spirit of parameter variation, interestingly, we also noted that increasing the time of exposure at 20-mT strength (at least to 30 min) did not achieve any greater effects than a 10-min exposure (data not shown); but ideally, robust temporal effects might best be studied at a field strength beyond that to which we were limited (20 mT) with our current equipment.

In our study, the use of FGF-2 as a probe for the presence of anionic (sulfated) moieties on HS chains is particularly important for suggesting relative charge density on the cell surface. We were specifically interested in charge density rather than the quantity of HS chains per se because HS molecules may vary in the degree of sulfation per chain, depending on the cell type (23). Along these lines, flow cytometry studies demonstrated a relatively high degree of HS-dependent FGF-2 binding on A549 cells, showing a dramatic sensitivity to H'ase-III mediated HS destruction (Fig. 1 *D*) as compared with hLEC cells (Fig. 3 *C*), and suggesting the importance of a relatively high density of HS-mediated anionic charge that makes A549 cells susceptible to magnetic induction. This might also be the case for other tumor cells that are known to overexpress HS or other sulfated GAGs, such as chondroitin sulfate, on the cell surface (i.e., as in Figs. 1 *A* and S2 for A549 and LLC tumor cells, respectively).

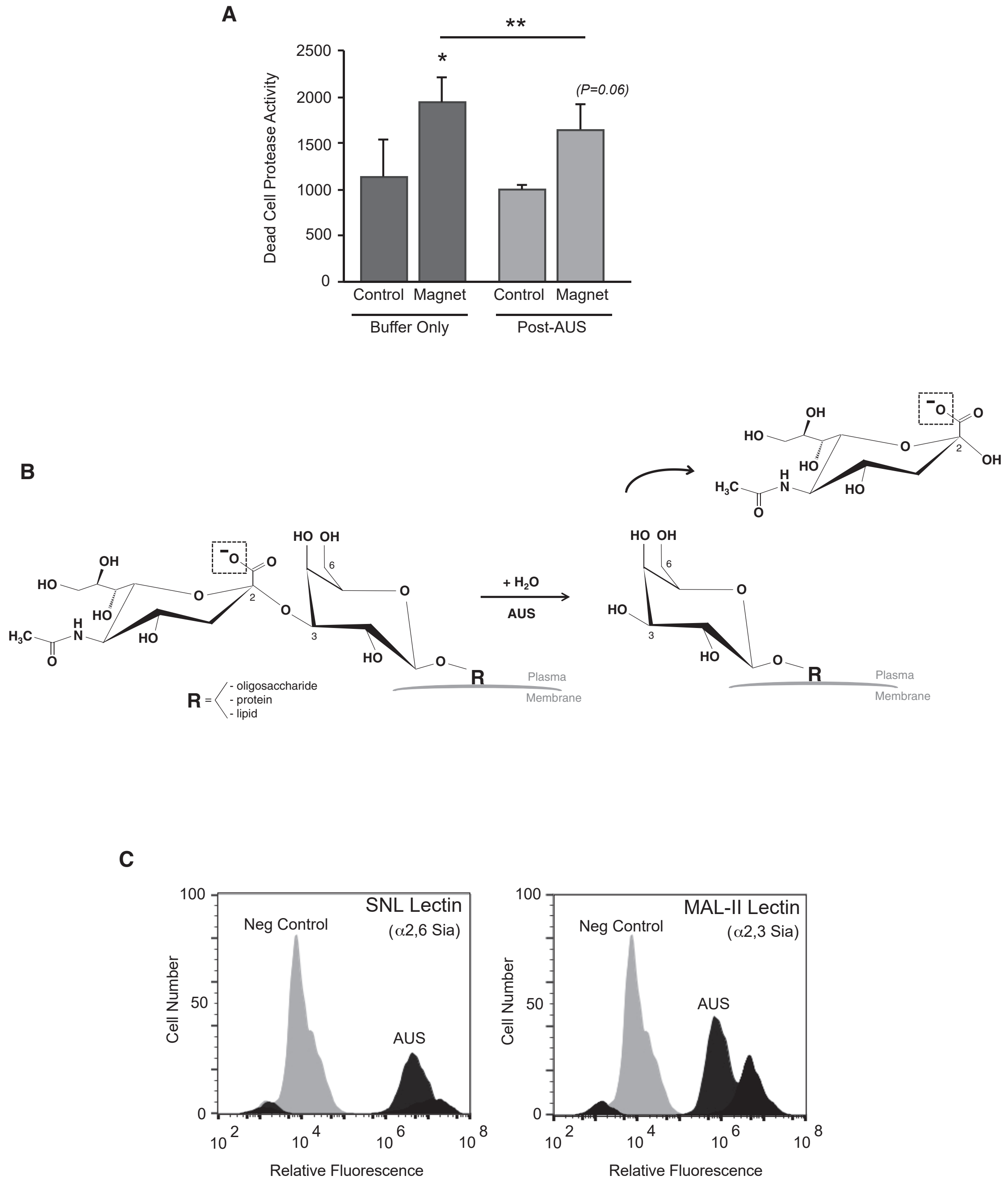

FIGURE 4 A pulsed magnetic field induces plasma membrane leak in MDA-MB-231 human breast carcinoma cells that is partially sialic acid dependent. (*A*) Membrane integrity from magnet-exposed and control MDA-MB-231 cells showing relative protease release signal ("Dead Cell Protease Activity") of control versus magnet-exposed cells on the graph (*left*, "buffer-only" bars, $*p = 0.02$ for difference in means by paired *t*-test; means represent average and SD among n = 3 experiments). In AUS-treated cells, magnet exposure also results in a protease release effect (*right*, "post-AUS" bars, $p = 0.06$ for difference in means by paired *t*-test), albeit more modest compared with effect on control (i.e., "buffer-only") cells. (Treatment of nonmagnet control cells with AUS sialidase does not significantly affect protease release; compare post-AUS control with buffer-only control.) With a specific focus on magnet-exposed cells, AUS treatment results in a partial, albeit significant, reduction in protease release compared with that of buffer-only control cells (compare rightmost post-AUS "Magnet" bar to second from left buffer-only "Magnet" bar; $**p = 0.02$ for difference in means by paired *t*-test). (*B*) The treatment of cells with AUS sialidase in these experiments is inspired by the fact that MDA-MB-231 cells are endowed with a relatively high cell surface sialic acid content, which confers negative charge on terminal sialic acid restudies that cap glycans expressed on cell surface oligosaccharides, proteins, and/or lipids at physiologic pH, as illustrated in the schematic showing the anionic oxygen species (*boxed*) on a typical terminal sialic acid residue (α2,3 linked to galactose; *left side* of diagram). AUS sialidase cleaves all possible linkages of sialic acid to internal galactose (α2,3 or α2,6) or even internal sialic acid (α2,8). (*C*) Demonstration of reduction in cell surface sialic acid in replicate AUS-treated MDA-MB-231 cells: flow cytometry was carried out using fluorescent lectins (SNL and MAL-II)

(legend continued on next page)

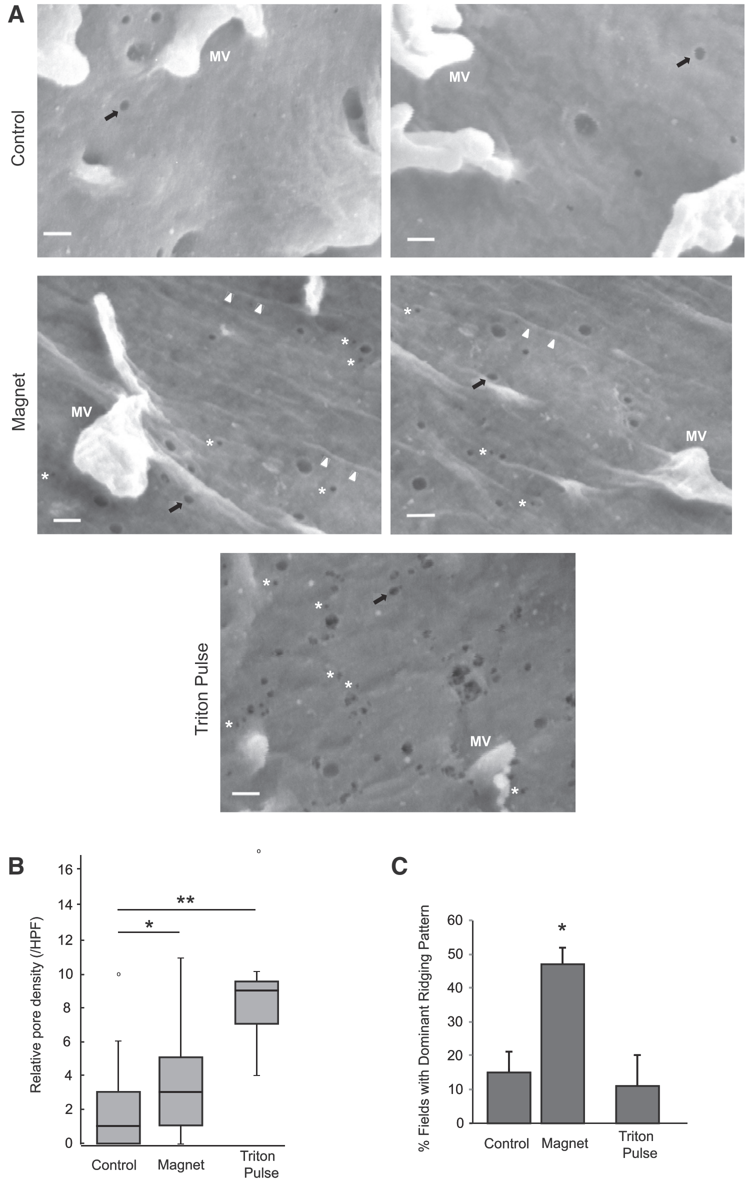

FIGURE 5 Regions of plasma membrane rippling and pores are induced by pulsed magnetic fields on A549 cells. (*A*) Scanning electron photomicrographs of the plasma membranes of control (*upper panels*) and magnet-exposed (*middle panels*) A549 cells immediately after exposure and fixation. The surface contains scattered microvilli (labeled MV in panels) as white protrusions; and several pores noted on both control cells and magnet-exposed cells include well-defined holes with diameters in the range of 20–50 nm (*black arrows*), consistent with likely caveolae or endocytotic events (bar = 100 nm). Smaller nanopores in the <20-nm-diameter range are highlighted (to *right* of *white asterisks*) on magnet-exposed cells. As an additional membrane-disruption control, the lower panel shows a photomicrograph of extensive membrane pore formation after a short exposure of control cells to a known detergent-based membrane-disruption agent (0.1% Triton × 1 min), demonstrating a high frequency of both small and larger pores (including presence of "moth-eaten" conglomerate holes). (*B*) Pores in the <20-nm size range (nanopores) were quantified in subfields of 600 × 600 nm within a series of random scanning electron photomicrographs taken from the surfaces of control cells (27 fields from n = 2 cells) and magnet-exposed cells (29 fields from n = 2 cells). The median (with interquartile range) value for number of nanopores per high-power field (HPF) is plotted on the graph, with the values for magnet-exposed cells (*middle bar*) and Triton-pulsed cells (*right-most bar*) normalized to the median for control cells (*far left bar*); $*p = 0.03$ for difference; Mann-Whitney U test one-sided; $**p < 0.001$ for difference. A unique finding in scanning electron photomicrographs from magnet-exposed cells is the relatively high frequency of fields showing parallel-running lines or ridging on the membrane (*white arrowheads*). (*C*) Such ridging was analyzed for all SEM fields (reported in *B* above) wherein the pattern occupied >50% of the field (i.e., "dominant" ridging pattern) by three independent, blinded observers. The mean frequency of such ridge-dominated fields (as percentage of total fields ± SD) for each condition is plotted; $*p = 0.03$ for difference in mean relative to control cells; paired *t*-test (and $*p = 0.05$ for difference in mean for magnet-exposed cells relative to that of Triton-pulsed cells).

Whether the decrease in membrane integrity is a result of lysis caused by a strong torsional Faraday-type induction force is difficult to know with certainty (24). Although this mechanism appears plausible, our findings may also prompt one to consider a number of possible downstream effects that could disrupt the tumor cell membrane. One possibility is direct membrane lipid bilayer disruption as a result of torsion on transmembrane proteins that are heavily modified extracellularly by negatively charged GAG chains. These have been well described as upregulated elements in carcinomas (10). Additionally, abnormal activation of voltage-gated ion channels may occur, for example through mechanical effects of proteoglycan motion on neighboring ion channels, along with activation of downstream second messenger systems (25). These mechanisms would require further studies beyond the scope of this work to be elucidated. Investigating the effects of narrowing pulse width (rise time for dB/dt) in the system may also be of interest because preliminary studies (data not shown) indicate that the greater protease leak effect lies at 50 Hz (rather than the higher 385 Hz) in this initial proof of concept,

to bind with terminal sialic-acid-expressing glycans on MDA-MB-231 cells. The graph on the left shows SNL binding (which depends predominantly on cell surface α2,6-linked sialic acid), comparing far-right SNL fluorescence curve (*black*) to negative control (*gray*) on the left: AUS treatment reduced SNL binding by >50% (note leftward shift in "AUS" curve on log scale). MAL-II binding (which depends predominantly on α2,3-linked sialic acid) was reduced nearly 5-fold (right graph; note more marked leftward shift in "AUS" curve relative to positive control).

which may be explained by the fact that the duty cycle of ∼10 ms for our low-frequency oscillating system can only “fit” within the period of the lower (50 Hz) frequency. Although testing narrower pulse widths to achieve higher EMFs at low field strengths would be of interest, as also suggested by Novickij and others (26) examining electroporation, detailed work using tumor-cell systems lies beyond the scope of this study and the capability of our current equipment. Importantly, we were able to observe the decrease in membrane integrity almost immediately after exposure to the magnet (within 15 min), indicating the possibility of early membrane-disrupting effects along with likely late second-messenger-mediated signaling pathway effects. Curiously, when we exposed mouse LLC tumor cells to the pulsed magnetic field, we found a similar altering effect on membrane integrity. Consistent with what is known about upregulation of GAGs in carcinoma, the membrane alteration phenomenon may imply an effect that is potentially generalizable to many other carcinoma subtypes.

Other negatively charged glycans, such as sialic acid, which “caps” terminal carbohydrate modifications that are overexpressed in several carcinomas (27), may also be susceptible to such EMF coupling. Indeed, we found that the susceptibility of a human tumor cell line that is rich in sialic acid expression (MDA-MB-231 breast carcinoma) to pulsed field effects at least partially depends on the presence of cell surface sialic acid (Fig. 4). This argues for the possibility of similar torsion or electromechanical coupling effects that might be mediated by induction EMF effects of the pulsed field on sialic-acid-capped glycoproteins on such cells, in a similar light to sulfated GAG considerations on proteoglycans. However, it is also intriguing to consider the possibility that proteoglycans, with a high degree of anionic charge on multiple sulfated glycan polymers per membrane-anchored core protein (illustrated in Fig. 1 *C*), may be susceptible to a relatively high EMF-coupled force, inducing torque about the protein anchor to the cell membrane (as compared to that of sialic-acid-modified glycoproteins, decorated with a much lower order of magnitude of anionic charge per protein). Sialic acid residues variably cap complex carbohydrates of typically O-linked mucin-rich glycoproteins in carcinomas. Thus, on a “per-molecule” basis, the overall contribution of HS proteoglycans to EMF-driven effects, for example, may be greater than that of sialic-acid-capped glycoproteins, but this may vary depending on relative abundances of each molecular type as well as the fine structure and degree of modifications. Indeed, it is intriguing that certain tumors produce abundant polysialic acid chains on their glycocalyces, which again may confer an especially high susceptibility to pulsed magnetic field effects.

More generally, a challenge worth addressing is whether protease release into the media as a result of the magnetic field exposure is caused by a phenomenon other than a physical, disruptive reduction in membrane integrity. However, this seems unlikely because measurements in the system were made immediately after exposure, and cellular processes that might lead to protease release and altered membrane integrity through other pathways, such as those that depend on induction of cellular expression pathways, would typically take hours to occur. We probed whether signs of physical disruption of the membrane might occur using scanning electron microscopy, intrigued also by a report that a distinct form of electromagnetic wave exposure employing 200-kHz tumor-treating electromagnetic fields applied for multiple hours to cultured glioblastoma tumor cells (28) results in “holes” in tumor cell membranes in the diameter range of 5–10 nm. Curiously we found an increase in the presence of holes in the <20-nm-diameter range, which we named nanopores, after pulsed field exposure (Fig. 5, *A* and *B*). In addition, we found lines of possible stress or ridging present on magnet-exposed cell membranes (Fig. 5 *C*): Whether such lines result from an EMF-driven “pull” or strain on the membrane as a result of electric coupling with charged GAG chains on membrane-anchored proteoglycans running on “lines of EMF” orthogonal to the pulsing magnetic field would require further dedicated study.

In addition to the membrane integrity effect, the short (10 min) pulsed magnetic field exposure was sufficient to affect A549 cell viability in both short-term (4 h) and longer-term (4 day) periods (Figs. 2, *A* and *B*). Short-term measurements of cell viability were conducted using an ATP production assay. The decrease in ATP production that we found in magnet-exposed cells reflects reduced viability through a change in metabolic activity. However, the decrease in overall metabolic activity, as reflected by ATP production, could be caused by the reduction of membrane integrity from the physical stimulus, which might alter viability through independent signaling pathways. It is our belief that future work may uncover the unique pathway(s) acting downstream of this, and to the best of our knowledge, a novel delivery of tumor cell specific electromechanical stress. Our initial tumor cell viability observations prompted us to examine the longer-term consequences of the pulsed magnetic field. We also found interesting effects of the single 10-min pulsed field exposure on cell viability, as measured by cell counts over a period of days in cell culture (Fig. 2 *B*). Furthermore, noncancerous hLEC cells showed no change in long-term growth after magnet exposure (Fig. 3 *B*), likely reflecting the lack of any detrimental effect of the pulsed magnetic field on their growth potential. Examining viability of tumor cells in three-dimensional spheroids exposed to pulsed fields, or how pulsed fields may affect viability of differentially labeled cocultured tumor and nontumor (stromal) cells would be informative in future developmental work carried out on its own merit. These observations may also welcome opportunities to manipulate magnetic fields with adjustments in existing systems, such as modified magnetic resonance or transcranial magnetic stimulation platforms. The length scales for such magnetic fields easily penetrate the human body, and incorporating the “pulsed

field" (dB/dt) interaction with unique molecular charge properties of tumor glycocalyx in the setting of these opportunities may allow for novel future therapeutic development.

CONCLUSIONS

In this proof-of-concept set of experiments, we exposed carcinoma cells to a pulsed magnetic field and found detrimental effects that follow a short exposure period, with immediate alterations to membrane integrity and subsequent viability. These cells, like other cancer cells, are known to heavily express charged glycans. Specific elimination of this charge rendered the cells insensitive to the magnetic field effect on the membrane. Furthermore, when applying the same pulsed magnetic field treatment and sequence of experiments to noncancerous cells (hLECs), no such membrane integrity or proliferation effects were found. This sequence of findings suggests that EMFs generated by a pulsed magnetic field on a densely charged tumor glycocalyx may result in tumor-selective effects on membrane integrity and cell growth. At the level of anion-rich glycans decorating membrane-anchored glycoproteins on the tumor glycocalyx, this may also imply the unique ability of pulsed magnetic fields to induce forces, including "torsion" on such molecules, with secondary effects on membrane integrity (Fig. 6).

The mechanistic considerations we report in this model set of studies may serve as a basis for future work that builds on our proof of concept, thus examining how variations in glycocalyx charge density, dB/dt rise time, pulse interval, and pulsed field exposure time may change the magnitude of the effects seen. It is notable that we could significantly alter both membrane integrity and tumor cell growth behavior as a result of an exposure that was only minutes in length, whereas prior studies often reported effects using exposures that lasted for hours or even days. Importantly, these experiments point to a possible selective therapeutic application of such fields in the setting of cancer.

SUPPORTING MATERIAL

Supporting Material can be found online at https://doi.org/10.1016/j.bpj.2020.02.013.

AUTHOR CONTRIBUTIONS

Most experiments were performed by C.P.A. with assistance from S.C.J. Data were collected and analyzed by C.P.A. with assistance from M.M.F. Conception of ideas and theoretical considerations along with experimental oversight and design were carried out by M.M.F. with C.P.A. at essentially all stages of the work from study inception to manuscript preparation. The manuscript was written by C.P.A. together with M.M.F., and methodology was supported and described with assistance from S.C.J., E.A., and W.C. Specifically, significant assistance with regard to magnetic field performance and characterization were provided by E.A., M.U., and W.C., and many theoretical discussions and experimental considerations were facilitated by assistance from J.F.

ACKNOWLEDGMENTS

We acknowledge Dr. T. Deerinck and Dr. M. Ellisman of the University of California San Diego National Center for Microscopy and Imaging

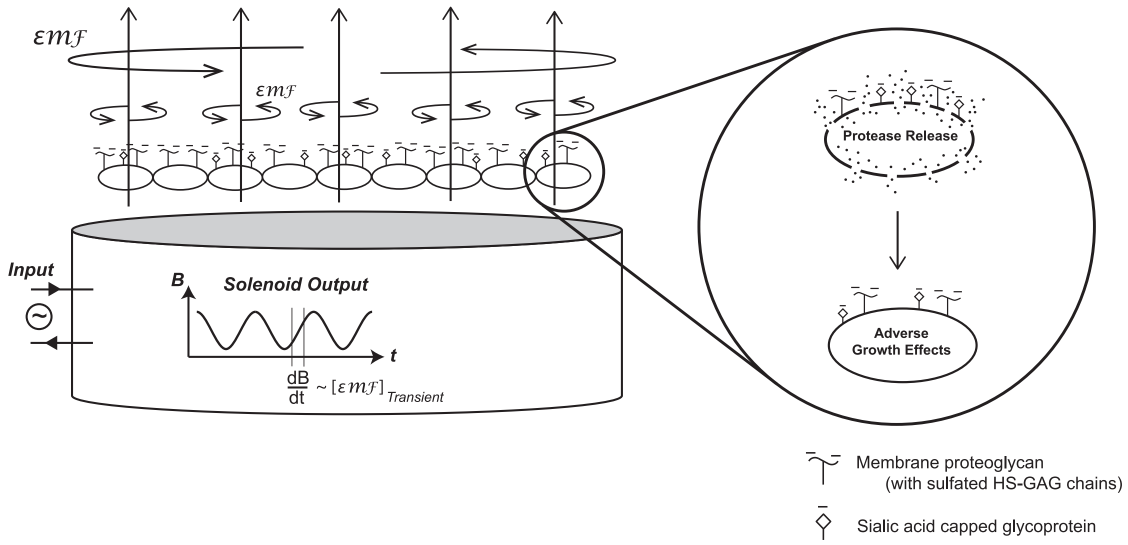

FIGURE 6 Diagram of experimental setup and potential magnetic field effects. A hypothetical cell layer with cartoon proteoglycans depicted on cell surfaces is shown above a magnetic platform from which circuit-driven pulsed B-field lines (shown pointing upward through the cell layer) are inducing EMFs, which may generate torsional forces on charged cell surface glycocalyx molecules. The latter may include anionic molecules, such as sulfated proteoglycans (τ-*shaped symbols* on cell surfaces) or sialic acid (*diamond shaped*) modifications that cap cell-surface glycoproteins, either or both of which are commonly upregulated in tumor cells. The EMFs may theoretically occur with variable strength depending on the bounded area through which the magnetic field fluxes as well as the magnitude of dB/dt. The magnified section shows potential biological effects on tumor cells, which appear from the findings herein to include membrane disruption and leak as well as altered viability.

Research (NCMIR) laboratory for assistance with scanning electron microscopy and image analysis. We also appreciate assistance (including use of heparin lyases and biotinylated FGF-2) from the laboratory of Dr. Jeffrey Esko.

We acknowledge funding from NIH/NHLBI (R01-HL107652 to M.M.F.; as well as P01-HL57345) and the U.S. Department of Veterans Affairs VA Merit Review Program (VA-Merit I01BX003688 to M.M.F.); and appreciate support from the Veterans Medical Research Foundation (M.M.F.). The work was also partly supported by a research grant from the W.M. Keck Foundation, USA (to J.F.), and performed as part of the San Diego Nanotechnology Infrastructure (SDNI) of UCSD, a member of the National Nanotechnology Coordinated Infrastructure, supported by the National Science Foundation (Grant ECCS-1542148 to J.F.) of the United States.

REFERENCES

1. Nie, Y., L. Du, …, T. Wang. 2013. Effect of low frequency magnetic fields on melanoma: tumor inhibition and immune modulation. *BMC Cancer.* 13:582.
2. Koh, E. K., B. K. Ryu, …, K. S. Chae. 2008. A 60-Hz sinusoidal magnetic field induces apoptosis of prostate cancer cells through reactive oxygen species. *Int. J. Radiat. Biol.* 84:945–955.
3. Williams, C. D., M. S. Markov, …, I. L. Cameron. 2001. Therapeutic electromagnetic field effects on angiogenesis and tumor growth. *Anticancer Res.* 21:3887–3891.
4. Omote, Y., M. Hosokawa, …, H. Kobayashi. 1990. Treatment of experimental tumors with a combination of a pulsing magnetic field and an antitumor drug. *Jpn. J. Cancer Res.* 81:956–961.
5. Tofani, S., D. Barone, …, F. Ronchetto. 2001. Static and ELF magnetic fields induce tumor growth inhibition and apoptosis. *Bioelectromagnetics.* 22:419–428.
6. de Seze, R., S. Tuffet, …, B. Veyret. 2000. Effects of 100 mT time varying magnetic fields on the growth of tumors in mice. *Bioelectromagnetics.* 21:107–111.
7. Tatarov, I., A. Panda, …, L. J. DeTolla. 2011. Effect of magnetic fields on tumor growth and viability. *Comp. Med.* 61:339–345.
8. Zhang, X., H. Zhang, …, W. Xiong. 2002. Extremely low frequency (ELF) pulsed-gradient magnetic fields inhibit malignant tumour growth at different biological levels. *Cell Biol. Int.* 26:599–603.
9. Kang, H., Q. Wu, …, X. Deng. 2018. Cancer cell glycocalyx and its significance in cancer progression. *Int. J. Mol. Sci.* 19:E2484.
10. Pinho, S. S., and C. A. Reis. 2015. Glycosylation in cancer: mechanisms and clinical implications. *Nat. Rev. Cancer.* 15:540–555.
11. Novikov, V. V., G. V. Novikov, and E. E. Fesenko. 2009. Effect of weak combined static and extremely low-frequency alternating magnetic fields on tumor growth in mice inoculated with the Ehrlich ascites carcinoma. *Bioelectromagnetics.* 30:343–351.
12. Nagarajan, A., P. Malvi, and N. Wajapeyee. 2018. Heparan sulfate and heparan sulfate proteoglycans in cancer initiation and progression. *Front. Endocrinol. (Lausanne).* 9:483.
13. Seidenfaden, R., A. Krauter, …, H. Hildebrandt. 2003. Polysialic acid directs tumor cell growth by controlling heterophilic neural cell adhesion molecule interactions. *Mol. Cell. Biol.* 23:5908–5918.
14. Storch, K., E. Dickreuter, …, N. Cordes. 2016. BEMER electromagnetic field therapy reduces cancer cell radioresistance by enhanced ROS formation and induced DNA damage. *PLoS One.* 11:e0167931.
15. Peterchev, A. V., T. A. Wagner, …, M. Bikson. 2012. Fundamentals of transcranial electric and magnetic stimulation dose: definition, selection, and reporting practices. *Brain Stimul.* 5:435–453.
16. Dechecchi, M. C., P. Melotti, …, G. Cabrini. 2001. Heparan sulfate glycosaminoglycans are receptors sufficient to mediate the initial binding of adenovirus types 2 and 5. *J. Virol.* 75:8772–8780.
17. Berry, L., M. Andrew, …, H. O'Brodovich. 1991. A549 lung epithelial cells synthesize anticoagulant molecules on the cell surface and matrix and in conditioned media. *Am. J. Respir. Cell Mol. Biol.* 4:338–346.
18. Yoshitomi, Y., H. Nakanishi, …, M. Okayama. 2004. Inhibition of experimental lung metastases of Lewis lung carcinoma cells by chemically modified heparin with reduced anticoagulant activity. *Cancer Lett.* 207:165–174.
19. Fuster, M. M., L. Wang, …, J. D. Esko. 2007. Genetic alteration of endothelial heparan sulfate selectively inhibits tumor angiogenesis. *J. Cell Biol.* 177:539–549.
20. Yayon, A., M. Klagsbrun, …, D. M. Ornitz. 1991. Cell surface, heparin-like molecules are required for binding of basic fibroblast growth factor to its high affinity receptor. *Cell.* 64:841–848.
21. Bertrand, R., E. Solary, …, Y. Pommier. 1994. Induction of a common pathway of apoptosis by staurosporine. *Exp. Cell Res.* 211:314–321.
22. Fuchs, S., A. J. Hollins, …, C. M. Lehr. 2003. Differentiation of human alveolar epithelial cells in primary culture: morphological characterization and synthesis of caveolin-1 and surfactant protein-C. *Cell Tissue Res.* 311:31–45.
23. Sarrazin, S., W. C. Lamanna, and J. D. Esko. 2011. Heparan sulfate proteoglycans. *Cold Spring Harb. Perspect. Biol.* 3:a004952.
24. Jiang, C., T. A. Lionberger, …, E. Meyhofer. 2016. Electromagnetic tweezers with independent force and torque control. *Rev. Sci. Instrum.* 87:084304.
25. Schwetz, T. A., S. A. Norring, …, E. S. Bennett. 2011. Sialic acids attached to O-glycans modulate voltage-gated potassium channel gating. *J. Biol. Chem.* 286:4123–4132.
26. Novickij, V., J. Dermol, …, D. Miklavcic. 2017. Membrane permeabilization of mammalian cells using bursts of high magnetic field pulses. *PeerJ.* 5:e3267.
27. Rodrigues, E., and M. S. Macauley. 2018. Hypersialylation in cancer: modulation of inflammation and therapeutic opportunities. *Cancers (Basel).* 10:E207.
28. Chang, E., C. B. Patel, …, S. S. Gambhir. 2018. Tumor treating fields increases membrane permeability in glioblastoma cells. *Cell Death Discov.* 4:113.

Biophysical Journal, Volume *118*

Supplemental Information

Pulsed Low-Frequency Magnetic Fields Induce Tumor Membrane Disruption and Altered Cell Viability

Christopher P. Ashdown, Scott C. Johns, Edward Aminov, Michael Unanian, William Connacher, James Friend, and Mark M. Fuster

SUPPORTING MATERIAL FOR:

Pulsed Low Frequency Magnetic Fields Induce Tumor Membrane Disruption and Altered Cell Viability

C.P. Ashdown[1,5] S.C. Johns[1,2] E. Aminov[6] M. Unanian[7] W. Connacher[6] J. Friend[6] and M.M. Fuster[1,2,3,4] *

[1] VA San Diego Healthcare System and [2] Veterans Medical Research Foundation, San Diego, CA, USA; [3] Department of Medicine, Division of Pulmonary & Critical Care, University of California San Diego, La Jolla, CA, USA; [4]Glycobiology Research and Training Center, University of California San Diego, La Jolla, CA, USA; [5] Division of Biological Sciences, University of California San Diego, La Jolla, CA, USA; [6]Dept of Mechanical and Aerospace Engineering, University of California San Diego, La Jolla, CA, USA; [7]School of Electrical Engineering, Columbia University, New York, NY, USA

SUPPLEMENTAL FIGURE LEGENDS

Supplemental Figure S1. Pulsed magnetic field exposure and photomicrograph of A549 monolayers showing density of cells at which exposure to magnetic fields was carried out. (A) A DC power supply was used to generate magnetic fields through a commercial solenoid in the milli-Tesla (20 mT maximum) range, with oscillating frequencies (50 Hz and 385 Hz) by interrupting the voltage input with an appropriate circuit for oscillations (shown to right). Cells plated as monolayers in wells of a 96-well plate (shown over the solenoid platform) were exposed to magnetic fields fluxing orthogonal to the plate-bottom. (B) View looking downward into plate-wells positioned over the solenoid platform, with configuration of 4 wells (marked in red) positioned over solenoid central-core (edge of uniform-grey metallic platform marked with a “P”) that uniformly exposed cells plated on the 4 marked wells to the orthogonal magnetic field emanating vertically from the central platform. (C) Typical appearance of sub-confluent A549 cells (at ~ 80% confluence in this photomicrograph) growing as monolayer within a well at time of pulse-field exposure. Bar represents 100 μm. Pre-experiment growth of all cells to approximately 80% confluence was carried out to ensure uniform and comparable monolayer conditions for membrane integrity and cell viability studies.

Supplemental Figure S2. Pulsed magnetic field associated reduction in membrane integrity of LLC tumor cells. Magnet-exposed and control LLC cells were measured using an intracellular-protease-leak detection assay, and graph shows mean protease release signal of magnet-exposed cells normalized to that of control cells (**P*=0.02 for difference between the means; paired T-test with means representing average and SD; n=5 experiments).

Supplemental Figure S3. Dose response plot showing relative magnet-induced protease release (normalized to max response) as a function of magnetic field amplitude. To assess for the relative degree of altered membrane integrity/ protease leak as it depends on magnetic field strength, 10 min exposures of A549 monolayers to sequential 50 Hz/ 385 Hz pulsed magnetic fields at room temperature were carried out, with measurement of dead-cell protease release values in magnet-exposed versus control cells. The increase in protease release by magnet-exposed cells over that of control cells (as a percentage of control protease release) was calculated and plotted for a scale of increasing magnetic field strengths, and normalized to the value at the maximum field strength (20 mT) achievable using the oscillating solenoid system illustrated in Supplemental Figure S1. The means +/- SD for n=3 experiments at each field strength are shown in the histogram (**P*=0.03; ***P*=0.01; One Sample T-test for positive magnet-induced protease release over that of control).

A

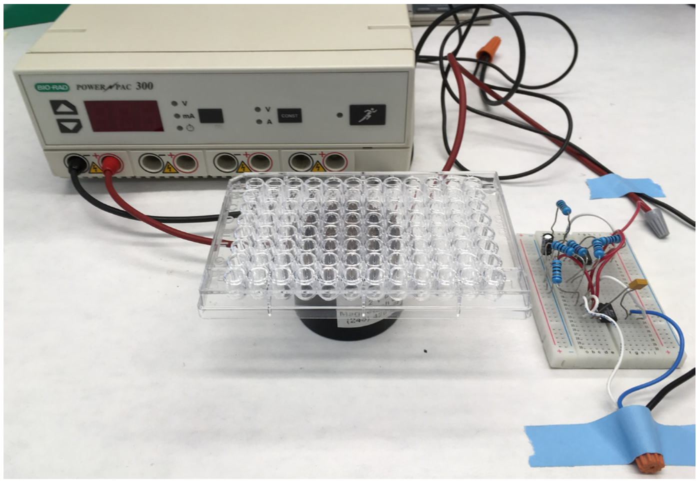

B

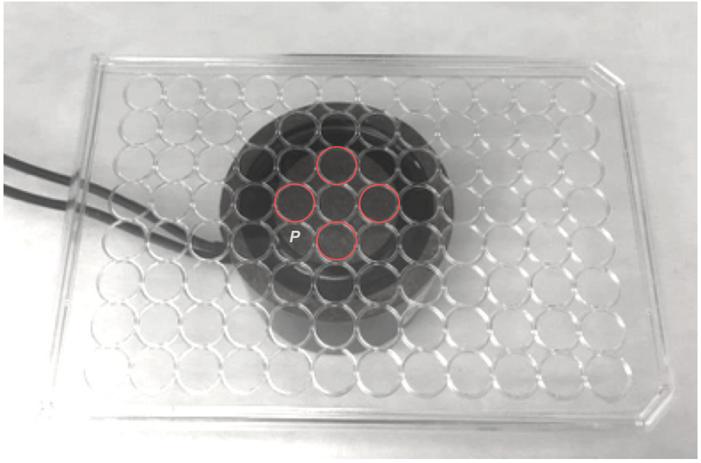

C

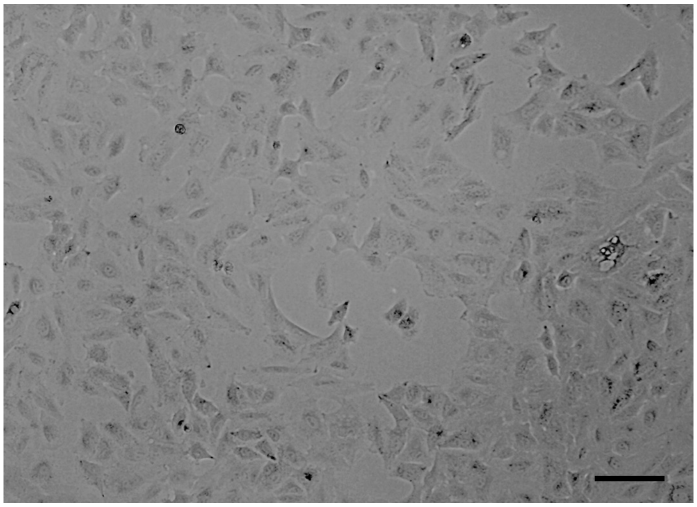

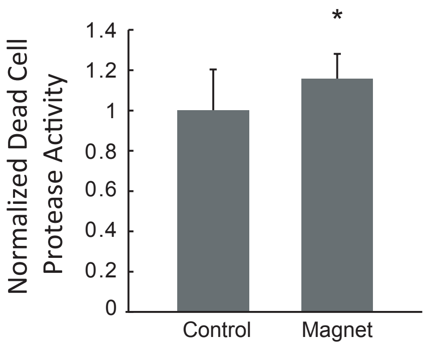
Normalized Dead Cell Protease Activity
1.4
1.2
1
0.8
0.6
0.4
0.2
0
*
Control
Magnet

Supplemental Figure S3

Ashdown, et al.

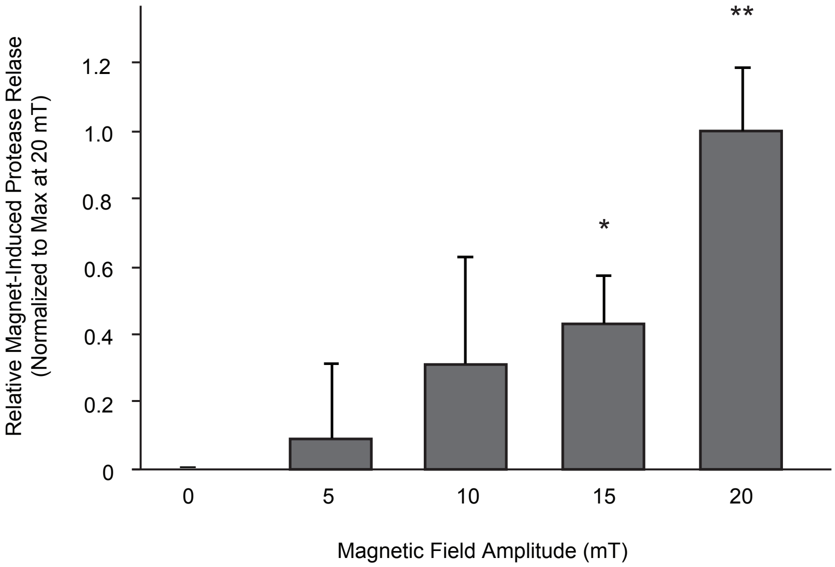

Supplement: Document S2. Article plus Supporting Material [file mmc2.pdf]
